# Physiological Corticosterone Attenuates gp120-Mediated Microglial Activation and Is Associated with Reduced Anxiety-Like Behavior in gp120-Expressing Mice

**DOI:** 10.3390/v15020424

**Published:** 2023-02-02

**Authors:** Emaya M. Moss, Fakhri Mahdi, Charlie J. Worth, Jason J. Paris

**Affiliations:** 1 Department of BioMolecular Sciences, School of Pharmacy, University of Mississippi, University, MS 38677-1848, USA; 2 Research Institute of Pharmaceutical Sciences, University of Mississippi, University, MS 38677-1848, USA

**Keywords:** affective behavior, glucocorticoids, glycoprotein 120, human immunodeficiency virus, microglia

## Abstract

Despite the benefits of combinatorial antiretroviral therapies (cART), virotoxic HIV proteins are still detectable within the central nervous system. Approximately half of all cART-treated patients contend with neurological impairments. The mechanisms underlying these effects likely involve virotoxic HIV proteins, including glycoprotein 120 (gp120). Glycoprotein-120 is neurotoxic due to its capacity to activate microglia. Corticosterone has been found to attenuate neuronal death caused by gp120-induced microglial cytokine production *in vitro*. However, the concentration-dependent effects of corticosterone on microglial activation states and the associated behavioral outcomes are unclear. Herein, we conducted parallel in vitro and in vivo studies to assess gp120-mediated effects on microglial activation, motor function, anxiety- and depression-like behavior, and corticosterone’s capacity to attenuate these effects. We found that gp120 activated microglia in vitro, and corticosterone attenuated this effect at an optimal concentration of 100 nM. Transgenic mice expressing gp120 demonstrated greater anxiety-like behavior on an elevated plus maze, and a greater duration of gp120 exposure was associated with motor deficits and anxiety-like behavior. Circulating corticosterone was lower in gp120-expressing males and diestrous females. Greater circulating corticosterone was associated with reduced anxiety-like behavior. These findings may demonstrate a capacity for glucocorticoids to attenuate gp120-mediated neuroinflammation and anxiety-like behavior.

## 1. Introduction

The human immunodeficiency virus type 1 (HIV-1) persists as a global pandemic, with approximately 36.7 million individuals living with the disease worldwide [1]. Combinatorial antiretroviral therapy (cART) has transformed HIV into a chronic-care illness. Although cART is efficacious at attenuating viremia peripherally, it is not well retained in the central nervous system (CNS). As such, approximately half of all people living with HIV (PLWH) contend with neurological impairments, collectively termed HIV-associated neurocognitive disorders (HAND) [2], irrespective of treatment with cART [3]. While cART has decreased the incidence of HIV-associated dementia, the most severe form of HAND, the incidence of neurocognitive impairment (i.e., asymptomatic and mild neurocognitive disorder) has remained consistent across the pre-cART and post-cART eras [3,4]. As a result, patients with HIV infection are at an increased risk of psychiatric illness, affective disorders such as generalized anxiety and major depression [5,6].

The mechanisms of HAND are not known, but likely involve the persistence of neurotoxic HIV proteins within the CNS. In particular, the HIV virotoxin, glycoprotein 120 (gp120) is present on the outer envelope of the HIV-1 virion and enables infection by facilitating fusion with the host cell via stepwise recognition of CD4 receptors and HIV co-receptors, predominantly CCR5 and CXCR4 [7,8,9]. Beyond its role in HIV entry, gp120 also promotes neurotoxic effects, partly via the activation of neuroinflammatory signaling mediated by chemokine co-receptors [8]. Activation of these receptors drives intracellular Ca2+ influx, mitochondrial dysfunction, and the production of reactive oxygen species (ROS), ultimately damaging or killing neurons [8]. Glycoprotein 120 also exerts indirect effects to promote neurotoxicity via the activation of monocyte-derived cells, such as microglia, the predominate reservoirs of HIV in the CNS. Soluble gp120 activates NF-κB, thereby increasing cytokines from immune cells [10,11,12]. These direct and indirect neurotoxic effects of gp120 may damage neurocircuits, impairing communication from the CNS to peripheral targets.

One such neurocircuit that may be impaired by gp120 involves the hypothalamic-pituitary-adrenal (HPA) stress axis. The HPA stress axis is activated by psychological, physical, and immune challenges [13,14] and is characterized by corticotrophin releasing hormone secretion from the hypothalamus to the anterior pituitary, which thereby secretes adrenocorticotropic hormone (ACTH) to promote glucocorticoid synthesis from the adrenals. This neuroendocrine signaling is tightly regulated, with glucocorticoids acting to attenuate their own production in a negative feedback loop. HIV patients experience HPA axis dysfunction, including hypercortisolemia (i.e., high basal cortisol levels) and adrenal insufficiency (i.e., paradoxically low cortisol in response to a stressor) [15,16]. Dysfunction of this neuroendocrine circuit may involve gp120. In support, mice that transgenically express gp120 are observed to have increased ACTH and circulating corticosterone [17]. Glucocorticoids are critical for psychological and immune homeostasis; they restore affective function and exert potent anti-inflammatory effects [18,19,20,21,22]. Moreover, corticosterone is found to attenuate the indirect effects of gp120 to promote neurotoxicity in vitro [23]. As such, we aimed to assess the ameliorative capacity of glucocorticoids on gp120-mediated neuroinflammation and the affective consequences of gp120 on HPA dysregulation in a whole animal model.

In order to assess the potential anti-inflammatory role that glucocorticoids may exert over HIV-1 gp120, the concentration-dependent effects of corticosterone to attenuate gp120-mediated activation of human microglial cells (HMC3) was assessed *in vitro*. To assess the potential effects of gp120 on anxiety- and depression-like behavior, transgenic mice that constitutively expressed the gp120 protein in a GFAP-relegated manner were tested in a behavioral battery of motor and affective responding. Circulating corticosterone was also assessed. We anticipated that HIV-1 gp120 would activate microglia and promote affective dysfunction, and that corticosterone would ameliorate these effects.

## 2. Materials and Methods

All procedures were preapproved by the Institutional Animal Care and Use Committee at the University of Mississippi. Experimental procedures were conducted in accordance with the National Institutes of Health Guide for the Care and Use of Laboratory Animals (NIH Publication No. 85-23).

### 2.1. In Vitro Determination of Microglial Activation

A human microglial cell line (HMC3, #CRL2266 from ATCC, Manassas, VA, USA) was used to assess the influence of gp120 and corticosterone activation. Cells were seeded onto 24-well plates at a density of 5000 cells/well. To avoid cell density bias, cells were seeded starting from the center of the well and pipetted clockwise. Cells were maintained in growth medium: 89.5% EMEM (Invitrogen Life Technologies, Carlsbad, CA, USA), 10% heat-inactivated fetal bovine serum (FBS; endotoxin concentration ≤ 25 EU/mL; Thermo Scientific Hyclone, Logan, UT, USA; Cat # SH30910.3; Lot # AG29485626), and 0.5% antibiotic (Gibco; Cat # 15240-062)/antimycotic (anti-anti 100×; Invitrogen Life Technologies; Lot # 2087430) mixture. Cells were seeded on day 1 and underwent experimental manipulations on day 2. On the day of the assay, media were fully exchanged with media containing corticosterone (0, 32, 100, or 320 nM; Sigma-Aldrich, St. Louis, MO, USA; Lot # 2087430) and treated with or without R5-tropic gp120_ADA_ (500 pM; ImmunoDx, Woburn, MA, USA; Cat # 1081). The concentration range of corticosterone was chosen to be within the low-to-high physiological range observed in the brain [24,25]. The concentration of gp120 was chosen given that it has previously been demonstrated to promote neuropathology in vitro [26,27,28,29]. All treatments were conducted in technical duplicate on each plate. Duplicates on each plate were averaged and were considered one observation. Each plate contained a negative control (media alone) and a positive control (gp120 alone). On independent plates, 2, 6, or 24 h after treatment, cells were fixed with 4% paraformaldehyde.

Immunocytochemistry: Fixed cells were permeabilized and blocked with blocking buffer (1% BSA, 1% normal goat serum in PBS), and followed by labeling with an antibody against ionized calcium-binding adaptor protein-1 (rabbit anti-Iba-1; 1:200; FUJIFILM Wako Pure Chemical Co., Richmond, VA, USA; #019-19741) overnight at 4C. The cells were treated with Texas Red goat anti-rabbit secondary antibody (1:500; Invitrogen Life Technologies, #T2767). Cells were counterstained with Hoechst 33342 nuclear stain (1:10,000; Invitrogen Life Technologies). Cells were imaged on a Nikon Ti-2E wide-field epifluorescent microscope with a motorized stage using a 20× objective (ELWD 20×/0.45; ∞/0.2 WD 8.2-6.9). One image was taken per well in the center of the well with one field deviation to the left or right. Approximately 22 cells per field were counted.

Microglial morphological assessment: To identify the activation state of microglia represented in each microscopic field, cellular morphology was scored as previously described with some modifications [30,31]. The scale from prior publications was simplified to differentiate only surveillant microglia, active/reactive microglia, or amoeboid/phagocytic microglia. The total number of cells (identified by Hoechst nuclear staining) was counted, and the proportion (%) of each cellular morphology was reported. ‘surveillant’ cells were identified by an elongated morphology with extended processes, ‘activate/reactive’ cells were identified by a ramified morphology with retracted processes, and ‘amoeboid/phagocytic’ cells were identified by a rounded amoeboid morphology. An activation scale was used to describe the average proportion of activation in each field. As such, cells with a surveillant morphology received a score = 1, active/reactive cells received a score = 2, and amoeboid/phagocytic cells received a score = 3 [32].

### 2.2. Subjects and Housing

HIV-1 gp120-transgenic female and male mice (6–12 mos. old; male gp120(−) mean age = 8 mos. old; male gp120(+) mean age = 7 mos. old; proestrous gp120(−) mean age = 10 mos. old; proestrous gp120(+) mean age = 10 mos. old; diestrous gp120(−) mean age = 9 mos. old; diestrous gp120(+) mean age = 9 mos. old) that did [gp120(+)] or did not [gp120(−)] express the gp120 transgene were generated in the vivarium at the University of Mississippi (University, MS). Mice cages were housed in a room with both male and females. Mice were fed Teklad 2018 mouse chow (Envigo; Indianapolis, IN, USA) and tap water. Initial breeders were obtained as a generous gift from Dr. Marcus Kaul (University of California at Riverside, Riverside, CA, USA) and are not commercially available. Transgenic gp120(+) mice express soluble X4-tropic gp120 (HIV-1 LAV) under the control of a glial fibrillary acidic protein promoter, as previously described [33]. Mice cages were housed with males and females from the same transgenic colony. All mice were gonadally-intact and housed in same-sex groups of 4–5/cage in a temperature- and humidity-controlled room on a reversed 12:12 h light/dark cycle (lights off at 09:00 h) with ad libitum access to food and water.

### 2.3. Determination of Estrous Cycle Phase

Estrous cycles were tracked by the daily collection of vaginal epithelia as previously described [34], with modifications to the sample collection time. Samples were collected at the start of the dark phase of the light cycle each day. Briefly, the diestrous phase is characterized by estradiol levels that are rising to peak and progesterone levels that are at nadir [35]. In the proestrous phase, estradiol levels are declining, and progesterone levels are at their peak [35]. Cycle phase was determined by morphology as previously described (diestrus was indicated by a majority presence of leukocytic cells, and proestrus was indicated by a majority presence of nucleated epithelial cells) [34].

### 2.4. In Vivo Behavioral Assessment

Female gp120 transgenic mice were behaviorally tested when in the proestrous or diestrous phases of their estrous cycle. Mice with irregular estrous cycles (no proestrous phase detected within two weeks; n = 5) were excluded from the study without testing. Each day of testing was counterbalanced between experimental groups. Male (gp120(−) n = 9; gp120(+) n = 9) and female (proestrous gp120(−) n = 9; proestrous gp120(+) n = 9; diestrous gp120(−) n = 9; diestrous gp120(−) n = 9) mice were acclimated to the behavioral testing room for 30 min prior to testing and were assessed approximately 1–3 h into the dark phase of their light cycle when the mice are the most active. Behavior was tracked and encoded by an ANY-maze behavioral tracking system (Stoelting Co., Wood Dale, IL, USA). All behavior apparatus was focally lighted at ~100 lux in the center. All mice were assessed in the behavioral battery described below in the order of open field, light dark transition test, elevated plus maze and tail suspension. After the completion of each test, mice immediately proceeded to the next without delay. We have previously found that testing in a similar behavioral battery does not produce notable carryover effects and does not influence steroid concentrations in plasma or brain [36]. Immediately following the completion of behavioral testing, mice were sacrificed by cervical dislocation by the experimenter who performed behavior via rapid decapitation in a separate room from behavior. The room mice were euthanized in was cleaned with ethanol in between each cohort.

Open field test: The open field test was used to assess spontaneous motor and anxiety-like behavior [37]. Briefly, mice were placed in a corner of a square open field box (40 × 40 × 35 cm^3^; Stoelting Co., Wood Dale, IL, USA) with a brightly lit center (inner 20 × 20 cm^2^) and allowed to behave for 5 min. Behavior was assessed for 5 min in order to capture spontaneous affective activity; habituation to this test produced decreased motor activity [36,38]. We have demonstrated this protocol to be sensitive to affecting behavior across rodent models [34,36,38,39]. Briefly, the distance (m) and velocity (m/s) traveled, as well as the frequency and times spent rearing, were used as indices of spontaneous horizontal and vertical motor behavior, respectively. The latency to enter, and the total time spent in, the brightly lit center of the maze was considered an index of anxiety-like behavior.

Light-dark transition test: Following testing in the open field, mice were immediately assessed for anxiety-like behavior in the light-dark transition test [40]. The light-dark apparatus consisted of two compartments, one brightly lit (20 × 20 × 35 cm^3^), the other dark and enclosed (20 × 20 × 35 cm^3^; Stoelting Co.). Briefly, mice were placed in a corner on the brightly lit side of the apparatus and permitted to behave for 5 min. The latency to enter the dark chamber and the total time spent in the light zone were considered indices of anxiety-like behavior. The total number of transitions between chambers was used as an index of motor behavior.

Elevated plus-maze: Immediately following the light-dark transition test, mice were assessed in the elevated plus-maze for anxiety-like behavior [41]. Briefly, the plus-shaped elevated apparatus (37.5 cm from the floor) consisted of two open and two enclosed arms (61 × 5 cm^2^) connected to a central area (5 × 5 cm^2^). Mice were placed in the central area facing one open arm and allowed to explore freely for 5 min. The latency to enter the open arms and the time spent on the open arms of the maze were considered indices of anxiety-like behavior. The number of total arm entries was recorded as an index of locomotor activity.

Tail suspension test: Immediately following the elevated plus-maze, mice were assessed for depression-like behavior in the tail suspension test [42,43]. Briefly, mice were suspended vertically, and their tails were secured with laboratory tape to a horizontal surface 18 in. above the floor. A small plastic cup was placed over the tail prior to taping to prevent tail-climbing. Behavior was recorded for 6 min (with the initial 2 min discarded for acclimation). The time spent immobile (i.e., adoption of a completely stationary posture with the exception of whole-body swaying from the momentum of prior movement) was quantified. The time spent immobile was considered an index of depression-like behavior

### 2.5. Circulating Steroid Assessment

Tissue collection: Immediately following the completion of behavioral testing, mice were sacrificed by cervical dislocation followed by rapid decapitation. Trunk blood was immediately collected in a chilled 1.5-mL aliquot tube, which was kept on ice until later centrifugation (13,500× *g*, 4 °C for 20 min). Serum was collected and stored in clean tubes at −80 °C until the time of the assay.

Steroid extraction: Circulating steroids were isolated from serum using ether-steroid extraction as previously described [42]. Briefly, serum samples were incubated with 1 mL of anhydrous ethyl ether and snap-frozen using a bath of dry ice/acetone. Supernatant was collected, evaporated to dryness in a fume hood overnight, and reconstituted to 50× the original volume in extraction buffer (Neogen Life Sciences, Lexington, KY, USA).

Enzyme-linked immunosorbent assay (ELISA): Circulating corticosterone was assessed via an ELISA kit per manufacturer instructions (Neogen Life Sciences; #402810) and as previously described [42]. For all assays, absorbance was read at 650 nm using a CLARIOstar microplate reader (BMG Labtech Inc., Cary, NC, USA).

### 2.6. Statistical Analyses

Microglial morphology and activation were assessed via repeated measures analyses of variance (ANOVAs) with treatment (i.e., media or gp120) and corticosterone concentration (i.e., 0, 32, 100, or 320 nM) as the between-subjects factors and time (2, 6, or 24 h) as the within-subjects factor. Behavioral measures and circulating corticosterone content were analyzed via separate two-way ANOVAs with sex condition (i.e., proestrous female, diestrous female, or male) and genotype [i.e., gp120(−) or gp120(+)] as between-subjects factors. Main effects were followed by Fisher’s protected least significant difference post hoc tests to determine group differences. Interactions were delineated via simple main effects and main effect contrasts with α controlled for family-wise error. All analyses were considered significant when *p* < 0.05.

## 3. Results

### 3.1. GP120 Activates, and Physiological Corticosterone Quiesces, Microglia In Vitro

**Figure 1:**
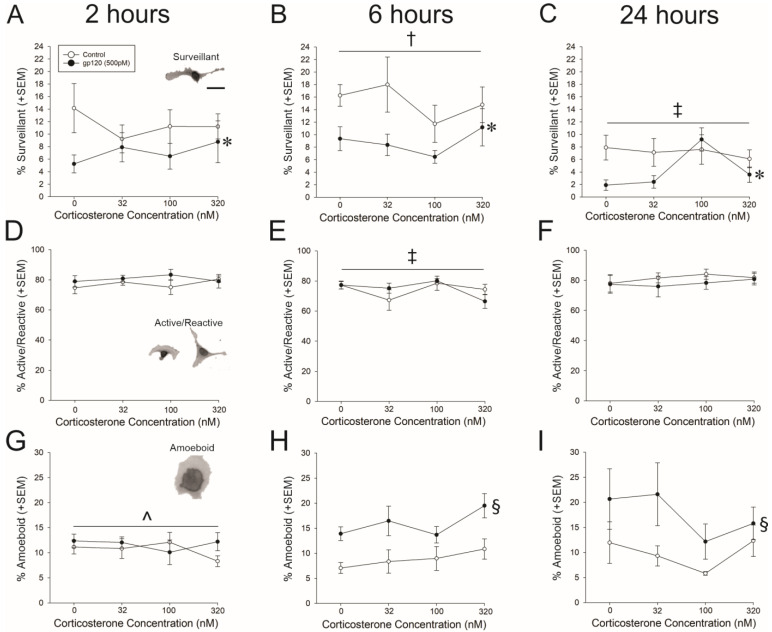
The proportion of surveillant microglia at 2 h (**A**), 6 h (**B**) and 24 h post-treatment (**C**). The proportion of active/reactive microglia at 2 h (**D**), 6 h (**E**) and 24 h post-treatment (**F**). The proportion of amoeboid/phagocytic microglia at 2 h (**G**), 6 h (**H**) and 24 h post-treatment (**I**). Figure (**A**,**D**,**G**) contain inset representative images of a surveillant, active/reactive, and amoeboid microglia, respectively (scale bare in A = 20 µm). † main effect of incubation time wherein 6 h significantly differs from 2 h; ‡ main effect time wherein the indicated time significantly differs from all other time-points; * main effect for gp120 to significantly differ from media control; § time × gp120 interaction wherein gp120 significantly differs from media control at 6 and 24 h only; ^ main effect of incubation time wherein the indicated time point is significantly different from all other time points; *p* < 0.05.

HMC3 cells were exposed to control media or gp120 (500 pM) with or without co-exposure to corticosterone (32, 100, or 320 nM) at 2, 6, and 24 h post-treatment. Gp120 significantly reduced the proportion of microglia that were surveillant, irrespective of time [*F*(1,110) = 20.33, *p* < 0.05], and there was a main effect for the proportion of surveillant cells to change over time [*F*(2,110) = 15.52, *p* < 0.05] (Figure 1A–C). After 6 h post-treatment (Figure 1B), significantly more cells exhibited a surveillance morphology compared to 2 h post-treatment (*p* = 0.01; Figure 1A). However, by 24 h, fewer cells exhibited a surveillant morphology than at any prior time-point (*p* < 0.0001–0.003; Figure 1C).

Neither treatment with gp120 nor corticosterone influenced the proportion of active/reactive cells (Figure 1D–F); however, significantly fewer cells exhibited an active/reactive phenotype at 6 h post-treatment [*F*(2,106) = 3.46, *p* < 0.05] (Figure 1E) compared to 2 h post-treatment (*p* = 0.04; Figure 1D) or 24 h (*p* = 0.01; Figure 1F).

Treatment with gp120 at different time significantly interacted to influence the proportion of microglia exhibiting an amoeboid morphology consistent with phagocytosis [*F*_Time X gp120_(2,108) = 3.38, *p* = 0.002] (Figure 1G–I). Compared to controls, gp120-treated cells exhibited a significantly greater proportion of amoeboid cells at 6 h and 24 h (but not 2 h; *p* < 0.0001–0.009; Figure 1G–I).

**Figure 2:**
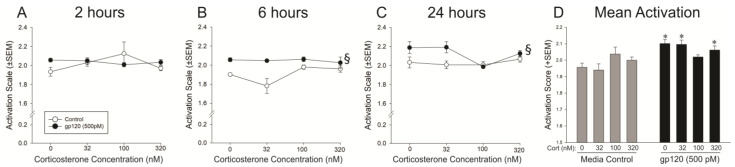
The average activation score of microglia at 2 h (**A**), 6 h (**B**) and 24 h post-treatment (**C**) and the mean activation score across all time-points (**D**). § time × gp120 interaction wherein gp120 significantly differs from media control at 6 and 24 h only; * gp120 × corticosterone interaction wherein gp120-exposure significantly increases activation of all microglia except those exposed to 100 nM corticosterone; *p* < 0.05.

Corticosterone concentration and gp120 exposure significantly interacted to alter the activation state of microglia [*F*(3,106) = 3.98, *p* < 0.05] (Figure 2). Irrespective of time, gp120 significantly increased the activation state of microglia compared to media control (*p* = 0.0008; Figure 2D). This was true even when microglia were co-exposed to gp120 and physiologically low (32 nM; *p* = 0.001) or high (320 nM; *p* = 0.01) concentrations of corticosterone (Figure 2D). However, microglial activation was attenuated by corticosterone when it was co-administered with gp120 at 100 nM (Figure 2D). The duration of exposure to gp120 and/or corticosterone also significantly influenced microglial activation state [*F*_Time X gp120_(2,106) = 3.37, *p* < 0.05; *F*_Time X Corticosterone_ (6,106) = 2.62, *p* < 0.05; Figure 2A–C]. Compared to the media control, gp120 significantly activated microglia to a greater degree by 6 h (Figure 2B) and 24 h (Figure 2C) post-treatment (*p* < 0.0001–0.003).

### 3.2. Duration of Exposure to gp120 and Sex Influence Anxiety-Like Behavior

Female and male mice that transgenically expressed (or did not express) gp120 were assessed for motor and affective performance in a behavioral battery. Sex differences were observed in motor/exploratory behavior in the open field test. Sex and genotype interacted such that proestrous females demonstrated a reduced frequency of rearing behavior compared to males [*F*_hormone cond X gp120 genotype_ (2,46) = 3.25, *p* < 0.05] (Table 1). Irrespective of genotype, females (proestrous and diestrous) also demonstrated significantly less rearing time compared to males [*F*(2,48) = 10.81, *p* < 0.05] (Table 1). No other differences in motor/exploratory behaviors were observed.

**Figure 3:**
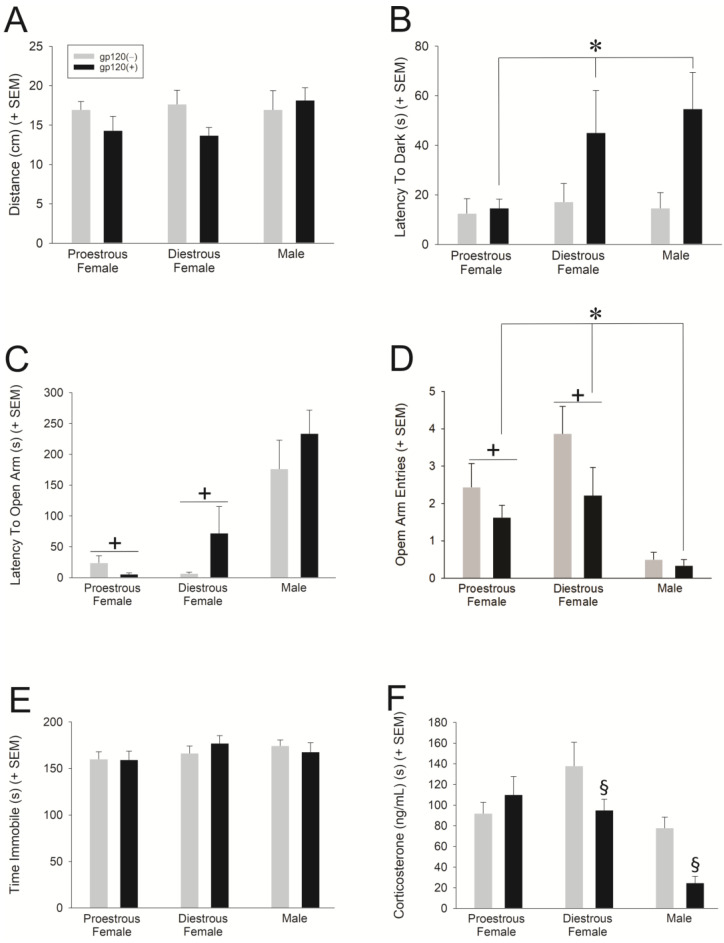
Mice were behaviorally assessed in an open field (**A**), light-dark transition task (**B**), elevated plus maze (**C**,**D**) and a tail suspension test (**E**). Circulating corticosterone was assessed from whole trunk blood (**F**). * main effect of genotype; + main effect of sex, wherein females are significantly different from males, irrespective of estrous cycle phase; § gp120 × sex interaction, wherein gp120(+) diestrous females and males significantly differ from their respective gp120(−) controls, *p* < 0.05.

Exposure to gp120 significantly increased anxiety-like behavior in the elevated plus maze. Irrespective of sex, gp120(+) mice made significantly fewer open arm entries in comparison to gp120(−) controls [*F*(2,45) = 12.6, *p* < 0.05] (Figure 3D). Unexpectedly, exposure to gp120 significantly decreased anxiety-like behavior in the light-dark transition test. Irrespective of sex, gp120(−) controls entered the dark zone significantly faster than did gp120(+) mice [*F*(1,48) = 7.59, *p* < 0.05] (Figure 3B). In addition, sex differences were observed on all assessments of anxiety-like behavior. In the open field, diestrous females made significantly fewer entries into the brightly-lit center of the maze than did males [*F*(2,48) = 3.12, *p* ≤ 0.05] (Table 1). In the light-dark test, a significant effect of sex was also observed, wherein proestrous females accumulated less time in the light zone [*F*(2,46) = 6.40, *p* < 0.05] and more time in the dark zone [*F*(2,48) = 3.67, *p* < 0.05] than did males (Table 1). In the elevated plus maze, male mice took longer to enter the open arms [*F*(2,46) = 21.7, *p* < 0.05] and made fewer open arm entries [*F*(1,45) = 4.15, *p* < 0.05] compared to proestrus or diestrus females, irrespective of genotype (*p* = 0.0001–0.021; Figure 3C,D). Compared to males, diestrous females spent more time in the open arms [*F*(2,46) = 3.36, *p* < 0.05] and proestrous females spent more time in the closed arms [*F*(2,47) = 3.68, *p* < 0.05] (Table 1). No significant depression-like behavior was observed in the tail suspension test (Figure 3E).

Older mice are expected to have a greater duration of gp120 exposure. As such, simple linear regressions between age (in days) and dependent measures were assessed (Table 2). Among gp120(+) mice only, older age negatively correlated with central entries in an open field, rearing time and frequency, and the distance and velocity traveled (Table 2). Contrary to expectation, the latency to enter and the proportion of entries into an open arm on an elevated plus maze were positively correlated with age (Table 2). Irrespective of genotype, light zone time was positively correlated with age (Table 2). Only among gp120(−) controls was the time spent immobile in a tail suspension test negatively associated with age.

### 3.3. Elevated Corticosterone Was Associated with a Decreased Anxiety-Like Response to gp120

Immediately following the completion of behavioral testing, blood was collected. Additionally, the circulating corticosterone level in serum was later assessed via lipid extraction and ELISA assay. Sex and genotype significantly interacted to influence corticosterone levels [*F*_hormone cond X gp120_ genotype (2,46) = 3.49, *p* < 0.05] (Figure 3F). Diestrous females and males that were exposed to gp120 had significantly lower circulating concentrations of corticosterone than did their respective gp120(−) controls (*p* = 0.01–0.045; Figure 3F). No such effect was observed among proestrous females (Figure 3F).

**Figure 4:**
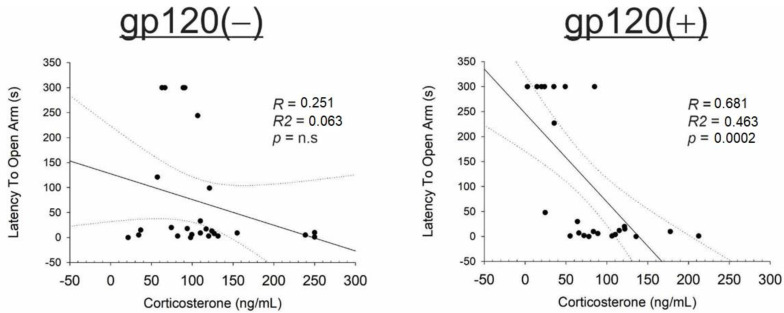
Simple linear regressions between circulating corticosterone among gp120(−) and gp120(+) females and males.

Circulating corticosterone levels were also analyzed via simple linear regression with dependent measures. Among gp120(+) mice, but not gp120(−) controls, circulating corticosterone negatively correlated with the latency to enter the open arms of an elevated plus maze (Figure 4), indicating reduced anxiety-like behavior when corticosterone is elevated. Notably, older age was also associated with greater circulating corticosterone in gp120(+) mice (*r* = 0.53, *R*^2^ = 0.28, *p* = 0.01), and this relationship was not observed in the gp120(−) controls.

## 4. Discussion

We anticipated that HIV-1 gp120 would activate microglia and that corticosterone would ameliorate these effects. Our in vitro findings support this hypothesis. At all time-points, gp120 reduced the proportion of cells exhibiting a surveillant morphology. Microglial activation interacted with time, as evidenced by an increase in the proportion of amoeboid/phagocytic cells at 6 and 24 h post-treatment. It was notable that 100 nM corticosterone appeared to restore the proportion of cells with a surveillance phenotype; however, this did not reach statistical significance. When all morphological change was assessed using an activation scale, only the 100 nM concentration of corticosterone significantly reduced activation to levels commensurate with control. Corticosterone at 32 or 320 nM concentrations did not ameliorate the activation effects of gp120. These findings are consistent with the past reports of gp120-mediated increases in cytokine production from mixed glial cells [44], as well as the capacity for corticosterone to attenuate these effects [23]. However, prior work utilized supraphysiological concentrations of corticosterone [23]; the current findings extend those of past reports to demonstrate that a mid-physiological concentration is optimal.

Given the neuroinflammatory effects of gp120 observed in cell culture, we anticipated that a whole-animal model of gp120 exposure would result in behavioral dysfunction, recapitulating a neuroHIV-like phenotype. Only modest behavioral deficits were observed among ~9-month-old gp120(+) mice, and some of these effects were moderated by sex and estrous cycle. In both female and male mice, exposure to gp120 increased anxiety on an elevated plus maze, as indicated by an increased latency to enter the open arms and a reduction in the frequency of open arm entries. It is notable that no differences were observed in the amount of time spent on the open arms, which is commonly seen with more profound anxiogenic stimuli, such as exposure to the HIV virotoxin, trans-activator of transcription (Tat) [45]. These data are consistent with prior observations that reported a modest increase in anxiety-like behavior among 6-month-old gp120(+) mice compared to controls [46]. The steroid hormone milieu may have contributed to the anxiety-like behavior observed. Among mice in the present study that were expected to have an estrogen-steroid hormone profile at nadir (i.e., diestrous females and males), circulating corticosterone was significantly lower, and anxiety-like behavior in the light-dark transition test appeared to be reduced; albeit, the latter observation did not reach statistical significance. These data are consistent with estrogen promoting vulnerability to gp120-mediated neuroHIV-like effects. In support, others have recently demonstrated females to be more vulnerable to the allodynic effects of intrathecal gp120 compared to males, and removal of the primary source of estrogens, the ovaries, attenuated this effect in females [47]. These potential steroid hormone interactions may influence HPA stress axis responsiveness. In the current study, elevated corticosterone levels were associated with reduced anxiety-like behavior in gp120-exposed mice. Thus, the potential for corticosterone to exert a protective effect over gp120-mediated neuroHIV-like behavior in mice is consistent with the protective effects observed in cell culture.

The duration of exposure to gp120 may be a critical predictor of the magnitude of neuroHIV-like pathology manifested. In the presently used gp120-transgenic mouse model, Cd83, Cd14, and IL-1β are significantly increased by 8 months of age and not at earlier time points (2 or 6 months of age) [48], and behavioral deficits including motor and spatial cognitive impairment have been found in older (12 mos. old), but not younger (3 mos. old) transgenic mice [48]. Similarly, 8–11-month-old gp120-expressing mice demonstrated impaired motor behavior and spatial learning concurrent with a perturbed electrophysiological profile in the hippocampus [49,50] as well as impairments in sensorimotor gating that were sex-dependent [51]. Among female gp120(+) mice, prepulse inhibition was impaired compared to control mice; however, this effect was not observed among males [51], consistent with the notion that estrogens confer vulnerability to the neuroHIV-like effects of gp120. However, gp120(+) males did exhibit a significantly increased startle response compared to any other group [51]. In our study, in order to maintain power in males, proestrous and diestorus gp120(−) or gp120(+) were included in the correlation analysis. The present findings extend these observations to demonstrate that age, which is indicative of gp120-exposure in this model, was a significant predictor of increased anxiety-like behavior in an open field. No relationship between age and anxiety-like performance was observed in gp120(−) control mice. As such, a greater duration of exposure to gp120 may promote neuropathology that produces measurable behavioral deficits later in life. One understudied neural pathway that may be affected by gp120 involves the HPA stress axis.

Approximately 20% of PLWH exhibit hypercortisolemia (i.e., increased basal cortisol), indicating a resting dysregulation of the HPA stress axis [52,53,54,55,56]. Moreover, these individuals suffer from paradoxical adrenal insufficiency in response to a stressor (i.e., blunted stress-provoked cortisol; [15,16,57]. The mechanisms of HIV-mediated HPA axis dysfunction are not known and may involve the actions of HIV virotoxins in the central nervous system. Previous studies have implicated HIV proteins, including Tat and viral protein R (Vpr), in these effects [45,58,59,60]. In particular, conditional-expression of Tat in the murine CNS increased hypothalamic corticotropin releasing factor (CRF) concurrent with increased basal corticosterone and adrenal insufficiency in response to a swim stressor [60]. Herein, we observe significantly lower circulating corticosterone in ~9-month-old gp120-exposed males and diestrous females compared to their control counterparts. These data extend prior reports of a transient corticosterone increases in gp120(+) mice that decline with age [17]. At 6- or 11-weeks of age, gp120(+) mice exhibited significantly greater basal corticosterone than did their gp120(−) counterparts [17]. However, by 6 months of age, circulating corticosterone had declined in the gp120(+) mouse such that it no longer significantly differed from controls [17]. The present findings reveal the continued course of basal HPA axis decline, to demonstrate that basal corticosterone is significantly reduced in comparison to gp120(−) controls, by 6–12 months of age. It is intriguing that this corticosterone decline was not observed in proestrous females. The activational effects of circulating steroid hormones may moderate some sex differences that are observed.

Clinical studies of HIV are rarely stratified by sex/gender [61], and even preclinical studies of gp120 have collapsed on sex as a factor [46]. However, sex may be an important predictor of outcomes. In the current study, circulating corticosterone was normalized in proestrous mice, an effect that could be indicative of the rise in potentially protective progestogen steroids. Progesterone is metabolized to a potent, neuroprotective steroid called allopregnalone (AlloP). Unlike progesterone, AlloP has virtually no affinity for nuclear progestin receptors [62] and instead acts as a potent positive allosteric modulator of GABA_A_ receptors [63]. In the brain, AlloP exerts rapid actions to downregulate CRF gene expression in the hypothalamus [64], reduce circulating glucocorticoid production [65,66], and to restore homeostasis as an endogenous modulator of the HPA axis. The protective effects of AlloP over gp120 have not been established but should be investigated given that AlloP is an FDA-approved therapy [67].

Activation of the HPA axis promotes the secretion of glucocorticoids, which are anti-inflammatory. However, cytokines also exert reciprocal effects on the HPA axis, creating a dynamic crosstalk between stress hormone and immune hormone signaling. Acutely, stress hormone production may be neuroprotective. In mixed glial cultures, gp120 promoted significant cytokine production that was neurotoxic [23,68]; however, corticosterone-treated media attenuated this effect [23]. *In vivo*, glucocorticoids promote phagocytic activation of monocyte-derived macrophages, contributing to the clearance of proinflammatory cells/debris and largely suppressing proinflammatory cytokine release [69,70]. However, these interactions between the HPA axis and the immune system are dynamic, and cytokines also exert effects on modulating glucocorticoid signaling via actions at glucocorticoid receptors (GR). When inflammation is chronic, GR function can be inhibited, thereby creating a state permissive for cytokine production [71,72]. The HIV protein, gp120, is an adequate stimulus to promote chemotactic, pro-, and anti-inflammatory cytokine production [73]. The molecular mechanisms by which cytokines may influence GR signaling are beginning to be explored. Thus, monocyte-derived cells within the brain present as both a target of glucocorticoid actions and a source of potential GR-mediating cytokines.

Together, these findings demonstrated the capacity for gp120 to promote microglial activation and for an endogenous glucocorticoid to attenuate these effects. In vivo exposure to gp120 promoted a modest anxiety-like phenotype in mice, and greater circulating concentrations of corticosterone were associated with a reduction of anxiety-like behavior on an elevated plus maze among those exposed to gp120. A greater duration of exposure to gp120 was associated with an exacerbation of neuro-HIV-like motor/affective impairment in an open field. Acting alone or in combination with additional HIV virotoxins, gp120 may play an important role in promoting neuroHIV and secondary effects on the HPA stress axis.

## Figures and Tables

**Table 1:** Anxiety-like behavior of female and male mice that expressed gp120 [gp120(+)] or did not [gp120(−)] (n = 8–9/group). Females were tested in either the proestrous or diestrous phase of their estrous cycle. * main effect of condition wherein proestrous females significantly differ from all other groups; **†** main effect of sex wherein the indicated group significantly differs from males; **‡** sex × genotype interaction wherein proestrous females significantly differ from males, *p* ≤ 0.05.

		Proestrous		Diestrous		Male	
		gp120(−)	gp120(+)	gp120(−)	gp120(+)	gp120(−)	gp120(+)
Open Field (5 min.)	Mean speed (m/s)	0.06 ± 0.003	0.05 ± 0.01	0.06 ± 0.01	0.05 ± 0.004	0.06 ± 0.01	0.06 ± 0.01
	Rearing Number	59 ± 7 **‡**	61 ± 9 **‡**	80 ± 15	53 ± 3	72 ± 5	91 ± 7
	Rearing time active (s)	49 ± 6 **†**	41 ± 5 **†**	59 ± 8 **†**	43 ± 3 **†**	92 ± 20	100 ± 19
	Center entries	12 ± 2	12 ± 2	10 ± 2 **†**	10 ± 2 **†**	16 ± 3	16 ± 1
	Center time (s)	17 ± 4	20 ± 3	11 ± 2	13 ± 3	17 ± 4	19 ± 2
	Latency to first entry (s)	26 ± 6	74 ± 27	35 ± 11	33 ± 8	36 ± 17	24 ± 4
Light-Dark Transition (5 min.)	Light zone entries	8 ± 1	10 ± 2	9 ± 1	10 ± 2	12 ± 3	8 ± 2
	Dark zone entries	8 ± 1	8 ± 2	9 ± 1	10 ± 2	38 ± 28	8 ± 2
	Light zone time (s)	46 ± 7 *	50 ± 10 *	100 ± 23	133 ± 25	92 ± 22	138 ± 28
	Dark zone time (s)	225 ± 27 *	231 ± 16 *	194 ± 22	162 ± 24	177 ± 29	157 ± 25
	% Light zone time (s)	20	22	52	82	52	88
	Distance (cm)	3 ± 0.7	4 ± 2	3 ± 0.5	3 ± 0.7	3 ± 0.8	4 ± 0.9
Elevated Plus Maze (5 min.)	Closed arm entries	10 ± 1	8 ± 1	12 ± 1	10 ± 1	10 ± 1	10 ± 1
	Open arm time (s)	11 ± 3	34 ± 15	39 ± 18 **†**	21 ± 10 **†**	6 ± 3	2 ± 1
	Closed arm time (s)	249 ± 12 **†**	227 ± 22 **†**	244 ± 8	246 ± 10	267 ± 7	270 ± 30

**Table 2:** Significant correlations between age (in days) and behavioral measures.

	gp120(−)			gp120(+)		
	*r*	*R^2^*	*p* Value	*R*	*R* ^2^	*p* Value
Center entries	−0.31	0.10	n.s	−0.35	0.12	0.08
Rearing Number	−0.25	0.06	n.s	−0.44	0.19	0.02
Rearing time active	−0.24	0.06	n.s	−0.51	0.26	0.01
Speed	−0.03	0.00	n.s	−0.50	0.25	0.01
Distance	−0.03	0.00	n.s	−0.49	0.24	0.01
Light Zone Time	0.51	0.26	0.007	0.38	0.15	0.05
Latency to Open Arm	0.28	0.08	n.s.	0.66	0.44	0.0002
% Open Arm Entries	0.23	0.05	n.s.	0.47	0.22	0.02
Time Immobile	−0.45	0.20	0.02	0.20	0.04	n.s

## Data Availability

Data are available upon request.
